# Cross-sectional comparison of individual and group psychosocial programs among individuals on probation with substance use problems in Türkiye

**DOI:** 10.3389/fpsyt.2026.1791526

**Published:** 2026-05-08

**Authors:** Utkan Boran Aşık, Samet Can Demirci, Zeynep Tomruk, Burak Erman Menkü

**Affiliations:** 1 Psychiatry Department, Faculty of Medicine, Lokman Hekim University, Ankara, Türkiye; 2 Türkiye Cumhuriyeti Adalet Bakanlığı, Ankara, Türkiye

**Keywords:** anxiety, depression, group intervention, hope, probation, self-efficacy, social support, substance use disorder

## Abstract

**Objective:**

This cross-sectional study aimed to examine whether participation in individual versus group-based psychosocial programs was associated with differences in love of life, sustained hope, cognitive flexibility, perceived social support, self-efficacy, addiction severity, and symptoms of depression and anxiety among individuals on probation who use substances.

**Materials and method:**

This cross-sectional, comparative study included one hundred participants who applied to the Ankara West Probation Directorate due to substance use and were placed on probation. Participants were assessed while enrolled in routine probation-based psychosocial programs, which are delivered over ten sessions in a duration of twenty weeks, either as individual interviews (n = 50) or as a group-based psychoeducation and counseling program (n = 50). Data collection involved a sociodemographic form as well as the Addiction Profile Index-Short Form (BAPI-Short), Love of Life Scale, Hope Scale, Cognitive Flexibility Inventory, Multidimensional Perceived Social Support Scale, Self-Efficacy Scale; and depression and anxiety subscales of Depression Anxiety Stress Scale–21. The two groups were compared using t-tests and chi-square tests for sociodemographic variables; analysis of covariance was used for psychosocial scales, with age, gender, education level, and income as covariates.

**Results:**

Participants in the individual and group programs were found to be similar in terms of age and basic sociodemographic characteristics. No significant difference was found in the total scores of the BAPI-Short, which indicates the severity of addiction. However, participants in the group program had significantly higher levels of love of life, sustained hope, cognitive flexibility, perceived social support, and self-efficacy, and significantly lower scores for depression and anxiety compared to those in the individual program. These differences persisted even after controlling for sociodemographic covariates and showed small-to-moderate effect sizes.

**Conclusion:**

In this cross-sectional sample, participants in the group-based program had more favorable psychosocial profiles, including higher levels of love of life, hope, cognitive flexibility, perceived social support, and self-efficacy, as well as lower depression and anxiety scores, than those in the individual program. These findings indicate an association between participation in group-based programs and more favorable psychosocial outcomes; however, due to the cross-sectional design, the absence of baseline assessments, and the possibility of selection bias, no causal conclusions can be drawn. Longitudinal and randomized studies are needed to clarify the direction and nature of these relationships.

## 1 Introduction

Substance use disorders are considered chronic mental health problems with a recurring course and significant consequences at both individual and societal levels. Substance use disorders continue to represent a substantial component of the global burden of disease, with more recent Global Burden of Disease analyses confirming their ongoing contribution to disability and premature mortality worldwide (1). The high rates of initiation of substance use, particularly during adolescence and young adulthood, lead to long-term negative consequences in many areas, including education, employment, family relationships, and legal issues.

Substance use disorders are seen at much higher rates in individuals who encounter the criminal justice system compared to the general population. Individuals on probation, in particular, are four to nine times more likely to develop substance use disorders, negatively impacting compliance with probation conditions, continuation of treatment, and the likelihood of recidivism (2). Substance use disorders are substantially overrepresented among people involved in the criminal justice system, and probation populations also show a high burden of co-occurring mental health problems that may complicate supervision, rehabilitation, and treatment engagement (3).

In Türkiye, the probation system aimed at including individuals, who have undergone legal proceedings due to substance use, has become an important mechanism in a community-based monitoring and treatment process. Studies conducted in Türkiye show that the vast majority of individuals on probation due to substance use are young adult males, a significant portion of whom live with their families and have regular employment (4, 5). In probation practice, individuals referred due to substance use are generally provided with semi-structured psychoeducation and counseling programs, typically twenty weeks long, consisting of one session per two weeks, and offered individually or in groups (5). Data from Türkiye show that individuals participating in these programs exhibit a heterogeneous structure in terms of substance use characteristics and sociodemographic profiles; however, the severity of substance use is high, and the age of seeking treatment is mostly concentrated in young adulthood (4, 5).

Studies conducted in Türkiye with individuals on probation have primarily focused on sociodemographic characteristics, substance use characteristics, and criminal history (5). However, studies examining psychosocial and mood-related areas such as love of life, hope, cognitive flexibility, self-efficacy, perceived social support, and depressive/anxious symptoms are limited. Yet these variables can play critical roles in both the continuation or cessation of substance use and in adaptation to the probation process and social functioning.

In the context of substance use disorders, these psychosocial variables can be understood as interrelated rather than isolated constructs. Perceived social support may provide an interpersonal context that facilitates hope, strengthens self-efficacy, and promotes more adaptive coping. In turn, higher hope and self-efficacy may be associated with greater persistence in recovery efforts, while cognitive flexibility may help individuals generate alternative responses to craving, stress, and interpersonal conflict. Depression and anxiety symptoms may undermine these recovery-related resources, whereas stronger psychosocial resources may coexist with more favorable emotional adjustment. For individuals on probation, who often face both legal pressure and psychosocial vulnerability, examining these variables together may offer a more comprehensive picture of recovery-related functioning than focusing on substance use severity alone.

The present study was guided by a recovery-oriented psychosocial framework, according to which change in substance use-related functioning is shaped not only by symptom severity but also by the interaction of interpersonal, cognitive, and emotional resources. Within this framework, perceived social support represents the relational dimension of recovery, hope reflects future orientation and goal-directed thinking, self-efficacy reflects perceived coping capacity, and cognitive flexibility reflects the ability to generate adaptive alternatives in response to stress and craving. Depression and anxiety, in contrast, may interfere with the effective use of these recovery-related resources. From this perspective, psychosocial programs may differ not only in format but also in the extent to which they are associated with interpersonal connectedness, reinforcement of adaptive beliefs, and emotional adjustment. This framework provided the conceptual basis for examining these variables together rather than as isolated outcomes.

Hope, self-efficacy, cognitive flexibility, perceived social support, addiction severity, and mood symptoms represent interrelated domains that are highly relevant to recovery in substance use disorders. Hope, conceptualized by Snyder et al. as a cognitive construct involving both goal-directed pathways and agency, has been associated with psychological adjustment, coping with adversity, and treatment adherence; in contrast, low hope may intensify helplessness and weaken recovery motivation (6). Similarly, self-efficacy, defined as the belief in one’s ability to successfully perform a behavior, is regarded as both a correlate of more favorable substance use outcomes and a clinically relevant mechanism of behavior change in addiction treatment, whereas low self-efficacy may increase reliance on substances in stressful or high-risk situations (7). Cognitive flexibility, referring to the capacity to generate alternative ways of thinking and behaving in response to changing demands, has also been linked to adaptive coping, lower mood and anxiety symptoms, and better adjustment; conversely, low flexibility may contribute to rigid thinking, limited coping alternatives, and greater relapse vulnerability (8). Perceived social support, encompassing emotional and instrumental support from family, friends, and significant others, is another important psychosocial resource, as higher support has been associated with lower depression and anxiety, better treatment adherence, and lower relapse risk in individuals with substance use disorders (9). In addition to these psychosocial domains, multidimensional assessment of addiction severity and emotional symptoms is also important. The Addiction Profile Index–Short Form (BAPI-Short), developed in Türkiye, provides a structured evaluation of substance use severity and related functional impairment and has been used in probation settings (5, 10), while the Depression Anxiety Stress Scale–21 (DASS-21) offers a brief and psychometrically supported measure of depression and anxiety symptoms, including in Turkish samples (11, 12). Taken together, these constructs provide a broader framework for understanding psychosocial functioning in individuals on probation with substance use problems beyond substance use severity alone.

Existing literature generally suggests that psychosocial resources such as perceived social support, hope, and self-efficacy are positively associated with recovery-related functioning in individuals with substance use disorders, while depression and anxiety tend to be associated with poorer adjustment. However, the literature is not entirely consistent regarding the relative importance of these factors or the contexts in which they are most salient. Some studies emphasize social support as a key determinant of treatment engagement and continuity, whereas others highlight the more significant role of internal resources such as self-efficacy or future-oriented cognitions such as hope. Similarly, although cognitive flexibility has been linked to adaptive coping and behavioral change, its specific role in probation-based populations remains less clear. These inconsistencies may reflect differences in study populations, treatment settings, outcome definitions, and methodological design. Therefore, rather than assuming a uniform pattern across settings, it is important to examine how these psychosocial variables cluster within specific service contexts such as probation, where legal coercion, social vulnerability, and treatment participation intersect.

The results summarized above demonstrate that individuals on probation should be assessed not only in terms of substance use severity but also in terms of multidimensional psychosocial domains such as love of life, hope, cognitive flexibility, self-efficacy, perceived social support, and depressive/anxious symptoms (13–15). Although individual and group-based psychosocial interventions are widely used in probation practices in Türkiye, studies comparing the possible different effects of these two approaches on these psychosocial variables are quite limited. In the current literature, only some studies have compared group interventions conducted within probation programs with motivational interviewing or individual interventions (15–17). The present study aimed to compare individuals who are on probation, use substances and participated in individual versus group-based psychosocial programs in terms of love of life, sustained hope, cognitive flexibility, perceived social support, self-efficacy, addiction severity, and symptoms of depression and anxiety. Rather than testing intervention effects, this cross-sectional study sought to identify whether these two naturally formed program groups differed on psychosocial and mood-related variables at the time of assessment.

Importantly, previous studies have not always distinguished whether these psychosocial factors should be conceptualized as predictors of recovery, correlations of current functioning, or potential consequences of treatment engagement. This ambiguity is especially relevant in cross-sectional probation research, where baseline differences between participants may influence observed psychosocial profiles. Accordingly, the present study does not assume that psychosocial program format causes these characteristics, instead it examines whether meaningful between-group differences are observable within a probation sample.

Based on this framework, we expected that participants in the group-based program would show more favorable profiles in psychosocial domains that are likely to benefit from interpersonal interaction and shared experience, particularly perceived social support, hope, and self-efficacy. We also explored whether these differences would extend to cognitive flexibility and mood symptoms. Given the limited and methodologically heterogeneous literature in probation settings, these expectations were treated as theory-informed but exploratory rather than strictly confirmatory.

## 2 Materials and methods

### 2.1 Participants

The study was conducted at the Ankara West Probation Directorate and included individuals on probation who are monitored under Article 191 of the Turkish Penal Code due to substance use. The sample was drawn from individuals who were already enrolled in routine psychosocial programs delivered at the directorate during the study period. Accordingly, the sampling strategy can be described as a single-center, non-random, consecutive clinical sample based on program attendance within routine probation practice. A total of one hundred participants were included in the analysis: Fifty were attending a program consisting solely of individual interviews, and fifty were attending a group-based psychoeducation and counseling program. Thus, the study compared two naturally formed program groups within the existing administrative and clinical structure of the probation service.

Inclusion criteria were: (i) being on probation under Article 191 of the Turkish Penal Code, (ii) regular attendance in one of the specified psychosocial programs during the study period, and (iii) provision of written informed consent. Individuals with known severe neurocognitive impairment, marked communication difficulties, or intellectual disability that would prevent meaningful completion of the scales were excluded. Participants who incompletely filled out the scales were excluded only from the relevant analyses.

### 2.2 Data collection tools

In addition to sociodemographic characteristics, various self-report scales were used in the study to assess addiction severity, hope, love of life, self-efficacy, perceived social support, cognitive flexibility, and depressive/anxious symptoms.

#### 2.2.1 Sociodemographic data form

This form was developed by researchers and used to determine participants’ basic demographic and clinical characteristics, such as age, gender, marital status, education level, employment status, income level, smoking and alcohol use, history of substance use, and previous treatment/probation history. These variables were controlled to define the sample and as potential confounders in statistical analyses (especially analyses of covariance).

#### 2.2.2 Addiction profile index-short form

The short form of the Addiction Profile Index was used to assess the severity of substance use. BAPI-Short is a 22-item, multidimensional self-report scale developed by Ögel et al. (10) to assess the level of substance use and various dimensions of addiction. The total score ranges from 0 to 44, with higher scores indicating more severe substance use.

#### 2.2.3 Hope scale

The Hope Scale, developed by Snyder et al. (6), was used to assess the level of hope. A validity and reliability study of the Turkish adaptation of the scale was conducted by Tarhan and Bacanlı, and the two-factor structure (Alternative pathways and Acting thought) was confirmed (18). The scale consists of twelve items, scored from 1 (“definitely false”) to 8 (“definitely true”), with a total score ranging from 8 to 64. Higher scores indicate a higher level of hope.

#### 2.2.4 Love of life scale

The Love of Life Scale was used to assess love of life and positive attitudes towards life. The scale was developed by Abdel-Khalek to measure the level at which life is perceived as an enjoyable and meaningful experience (19). The Turkish adaptation was carried out by Yıldırım and Özaslan, demonstrating the scale’s single-factor structure and high internal consistency coefficient (20). The scale consists of items rated from 1 (“no”) to 5 (“too much”); the total score ranges from 16 to 80, with higher scores reflecting a greater love of life.

#### 2.2.5 Self-efficacy scale

Individuals’ perceptions of their competence in coping with challenging life situations and achieving their goals were measured using the SES developed by Sherer and Adams and adapted into Turkish by Gözüm and Aksayan (21). (22). The scale is a 23-item, 5-point Likert-type self-report instrument (1 = “does not describe me at all”, 5 = “describes me very well”). The scale, which also includes reverse-scored items, yields a total score ranging from 23 to 115, with higher scores indicating a higher perception of self-efficacy.

#### 2.2.6 Multidimensional perceived social support scale

The Multidimensional Perceived Social Support Scale, developed by Zimet et al. (9), was used to assess the level of perceived social support. The scale consists of twelve items measuring perceived support from family, friends, and a “special person,” and is scored from 1 (“disagree”) to 7 (“strongly agree”). The Turkish adaptation and psychometric studies were conducted by Eker and Arkar, and the scale was reported to have good validity and reliability (23, 24). The total score ranges from 12 to 84, with higher scores indicating high perceived social support.

#### 2.2.7 Cognitive flexibility inventory

The level of cognitive flexibility was assessed using the Cognitive Flexibility Inventory developed by Dennis and Vander Wal and adapted into Turkish by Gülüm and Dağ (8, 25). The inventory consists of twenty items grouped into two sub-dimensions, “Alternatives” and “Control,” and is scored between 1 (“not at all appropriate”) and 5 (“completely appropriate”). Some items are reverse-scored; as the total score increases, cognitive flexibility is considered to increase.

#### 2.2.8 Depression anxiety stress scale–21

Depressive and anxious symptoms were assessed using the 21-item short form of the DASS, developed by Lovibond & Lovibond (26). A validity and reliability study of the Turkish short form of the DASS-21 was conducted by Yılmaz et al., and good internal consistency values ​​were reported for the three sub-dimensions (27). The scale consists of twenty-one items, each scored between 0 and 3. In this study, only the depression and anxiety subscales were used, and the relevant items were summed to obtain the DASS_Depression_Total and DASS_Anxiety_Total scores; higher scores indicate more severe symptoms.

### 2.3 Description of the program exposure

Within the routine probation system, psychosocial programming for substance-related probation cases was delivered in a semi-structured format. In line with standard probation practice in Türkiye, the programs typically extended over approximately twenty weeks and were delivered as one session per two weeks. In the present study, participants had been enrolled in one of two routine formats: an individual interview-based program or a group-based psychoeducation and counseling program.

The individual format consisted of scheduled one-to-one sessions conducted by the professional responsible within the probation service. These sessions generally focused on psychoeducation regarding substance use, motivation for change, treatment adherence, risk awareness, coping with triggers, and problem-focused counseling tailored to the participant’s needs.

The group format consisted of structured psychoeducation and counseling sessions delivered to groups of individuals on probation, consisting of 4–16 people. In routine practice, group sessions included information on substance use and its consequences, motivation and recovery, recognition of risky situations, coping skills, emotional awareness, interpersonal sharing, and discussion of strategies that may support adaptation to probation and treatment processes.

### 2.4 Procedure/ethics

The study employed a cross-sectional, comparative design and was conducted within the routine process of probation services. Before data collection, ethical approval was obtained from the Lokman Hekim University Scientific Research Ethics Committee, by the meeting number 2025/02, decision number 1, and decision date 28.02.2025; and institutional permission was obtained from the relevant probation authority. The study was conducted in accordance with the Declaration of Helsinki and relevant local regulations.

Eligible individuals on probation were informed both verbally and in writing about the purpose of the study, the voluntary nature of participation, the confidentiality of their responses, and the fact that participation or refusal would not affect their probation procedures or access to routine services. Written informed consent was obtained from all participants prior to data collection.

Program membership was determined from routine probation records, and participants were classified according to the psychosocial format in which they were already enrolled at the time of assessment; no allocation or reassignment was performed for research purposes.

Data collection was carried out in interview rooms available at the directorate. Participants first completed the Sociodemographic Data Form and then completed the BAPI-Short, HS, LLS, SES, MPSS, CFI, and the depression and anxiety subscales of the DASS-21 under supervision. For participants who are unable to read or write, the items were read aloud by the researcher and responses were recorded according to the participant’s statement. Program type (individual vs. group-based) was verified through probation files and program records and coded accordingly in the dataset.

### 2.5 Statistical analysis

Data were analyzed using SPSS 27. Mean and standard deviation (Mean ± SD) were calculated for continuous variables, and number and percentage [n (%)] were calculated for categorical variables. Sociodemographic characteristics of individual and group program participants were compared using independent samples t-test for continuous variables and chi-square (χ²) test for categorical variables.

Prior to conducting the analyses, statistical assumptions were examined. The dataset contained no missing values. The homogeneity of variance assumption was assessed using Levene’s test. The results indicated that this assumption was violated for several dependent variables. However, given that the group sizes were equal across conditions (n = 50 per group), the analyses were retained. Procedures based on the general linear model, such as ANOVA and ANCOVA, are generally considered robust to moderate violations of the homogeneity of variance assumption when sample sizes are equal across groups (28, 29). Therefore, ANCOVA was deemed appropriate for the present analyses.

The assumption of homogeneity of regression slopes was tested by including interaction terms between the grouping variable and each covariate in the model. None of these interaction terms were statistically significant, indicating that the assumption was satisfied.

## 3 Results

As shown in **Table 1**, the sample consisted predominantly of male individuals on probation, with a mean age of 28.31 ± 7.54 years; most participants were high school graduates, employed, and within the lower income categories.

**Table 1:** Sociodemographic data.

Variable	Category	n	%
Gender	Female and Other/Unspecified	5	5.0
	Male	95	95.0
Marital status	Single	63	63.0
	Married	25	25.0
	Divorced	11	11.0
	Other	1	1.0
Educational status	Unable to read or write, or Primary School	10	10.0
	Middle School	25	25.0
	High School	50	50.0
	University	15	15.0
Employment status	Employed	82	82.0
	Unemployed	14	14.0
	Student	4	4.0
Income status (per month)	≤22104,67 ₺	45	45.0
	Between 22104,67 ₺ and 44209,34 ₺	39	39.0
	More than 44209,34 ₺	16	16.0
Tobacco consumption	Yes	92	92.0
	No or Cessation	8	8.0
Alcohol consumption	Yes	46	46.0
	No	38	38.0
	Abstinence	16	16.0

Data are presented as mean ± SD for continuous variables and n (%) for categorical variables. SD, standard deviation.

**Table 2** presents the adjusted comparisons between the individual and group programs after controlling for age, gender, education, and income. No significant between-group difference was observed for BAPI-Short scores, F(1,94) = 0.27, p = 0.607, ηp² = 0.003. Significant between-group differences were found for LLS, F(1,94) = 6.81, p = 0.011, ηp² = 0.068; HS, F(1,94) = 11.56, p < 0.001, ηp² = 0.110; MPSS, F(1,94) = 14.26, p < 0.001, ηp² = 0.132; CFI, F(1,94) = 5.06, p = 0.027, ηp² = 0.051; SES, F(1,94) = 11.62, p < 0.001, ηp² = 0.110; DASS Anxiety, F(1,94) = 15.66, p < 0.001, ηp² = 0.143; and DASS Depression, F(1,94) = 22.69, p < 0.001, ηp² = 0.194. Adjusted mean differences were in favor of the group program for LLS, HS, MPSS, CFI, and SES, whereas DASS Anxiety and DASS Depression scores were lower in the group program.

**Table 2:** Differences between two groups according to research variables (controlling for age, gender, education, and income).

Scale	Individual (n=50) Mean ± SD	Group (n=50) Mean ± SD	F (1,94)	p	ηp²
BAPI-Short	8.34 ± 8.24	9.60 ± 9.44	0.27	0.607	0.003
LLS	59.30 ± 18.40	68.74 ± 14.76	6.81	0.011	0.068
HS	60.62 ± 20.52	72.26 ± 9.24	11.56	<.001	0.110
MPSS	44.26 ± 22.64	62.08 ± 19.80	14.26	<.001	0.132
CFI	69.18 ± 25.47	80.54 ± 11.49	5.06	0.027	0.051
SES	78.94 ± 19.84	88.52 ± 10.80	11.62	<.001	0.110
DASS Anxiety	7.26 ± 5.13	3.44 ± 3.41	15.66	<.001	0.143
DASS Depression	7.72 ± 5.36	3.34 ± 3.13	22.69	<.001	0.194

Values for the individual and group programs are presented as mean ± SD. BAPI-Short, Addiction Profile Index–Short Form; LLS, Love of Life Scale; HS, Hope Scale; MPSS, Multidimensional Perceived Social Support Scale; CFI, Cognitive Flexibility Inventory; SES, Self-Efficacy Scale; DASS Anxiety, Depression Anxiety Stress Scale–21 Anxiety subscale; DASS Depression, Depression Anxiety Stress Scale–21 Depression subscale; SD, standard deviation; F, F statistic; p, probability value; ηp², partial eta squared. ANCOVA models were adjusted for age, gender, education, and income.

## 4 Discussion

This study examined the association between participation in individual versus group-based psychosocial programs and psychosocial variables among individuals on probation who use substances within the scope of Article 191 of the Turkish Penal Code. The main finding was that, although the two groups did not significantly differ in addiction severity, participants in the group program reported higher levels of love of life, sustained hope, cognitive flexibility, perceived social support, and self-efficacy, as well as lower levels of depression and anxiety. These differences remained significant after controlling for age, gender, education, and income, and generally showed small-to-moderate effect sizes. However, given the cross-sectional design and lack of baseline data, these findings should be interpreted as between-group differences rather than evidence of program effects.

The findings may be interpreted within the recovery-oriented psychosocial framework. In this framework, recovery-related functioning is understood as involving both interpersonal and intrapersonal resources. The more favorable scores observed in the group program on perceived social support, hope, and self-efficacy are broadly consistent with the idea that group settings may be associated with shared identification, mutual encouragement, and social learning processes. At the same time, the findings also suggest that emotional and cognitive domains may be linked to these psychosocial resources, as participants in the group program also reported lower depression and anxiety and higher cognitive flexibility. However, given that the study was cross-sectional, these results should be interpreted as patterns of association rather than evidence that program format directly produced these differences.

International literature demonstrates that group therapy is a common and effective intervention method for substance use disorders. A meta-analysis examining group therapy for substance use disorders in adults reported that group psychotherapy generally provides significant benefits as an adjunct to treatment or compared to waiting list/without treatment conditions; and generally, shows similar levels of effectiveness compared to individual therapy (30). Similarly, studies systematically reviewing group-based substance use treatments highlight that cognitive-behavioral group programs, motivational groups, and contingency management groups are effective in reducing substance use across numerous substance types; and emphasize that group therapy offers significant advantages, particularly in terms of cost-effectiveness and peer support (31). While our results did not reveal a significant difference in substance use severity (BAPI-Short), the higher psychosocial well-being of individuals participating in the group program, consistent with this literature, points to the supportive and empowering dynamics of the group environment.

A key point in this study is that the sample consists of individuals on probation. Studies in the literature on this group, particularly group interventions, point to positive effects on criminal behavior and adjustment. Early work suggested that group formats may support behavioral adjustment among individuals on probation, but contemporary evidence specific to probation-based psychosocial programming remains limited. For example, in the classic study by Wagoner and Piazza with individuals on probation who use substances, it was reported that individuals on probation who participated in group therapy exhibited more “positive behavior” during the follow-up period (32). In an experimental study from Türkiye, it was found that psychosocial group interventions conducted with individuals who use substances significantly increased intrinsic treatment motivation and “confidence-in-treatment,” but did not create the expected lasting increase in self-efficacy perception (33). Our results, showing that individuals participating in the group program had higher levels of self-efficacy compared to those participating in the individual program, present a picture that is partly parallel to and partly more positive than that in the literature. This difference may be related to the structuring of group sessions in the context of probation, the number of sessions, and the relative intensity of their content.

The significantly higher level of self-efficacy found in the group program in our study is closely consistent with the addiction treatment literature. Self-efficacy has been identified as both a predictor of outcomes and a mediator of treatment effects in substance use disorder treatment (34). Many studies have shown that individuals with high self-confidence regarding substance use report less use after treatment and are more resistant to relapse. Furthermore, recent studies show that self-efficacy can affect not only substance use but also craving levels, along with areas such as self-esteem, loneliness, and social perception (35). In this context, the detection of higher self-efficacy levels in individuals participating in the group program can be considered a protective factor that reduces the risk of future relapse.

Similarly, perceived social support and, consequently, lower levels of depression and anxiety offer clues about the possible mechanisms of group programs. Social support is identified as a critical factor in addiction treatment, both in terms of treatment participation and long-term recovery. Several studies have shown that individuals with stronger social support networks stay in treatment longer and achieve better recovery outcomes (36, 37). A recent study revealed that mediating variables such as exercise self-efficacy and health-related quality of life may play a role in the relapse-reducing effect of social support (38). Due to the nature of group programs, greater interaction among participants, peer support, and a sense of belonging are expected; those can contribute to both increased perceived social support and decreased depressive/anxious symptoms.

It is noteworthy that in our study, hope and love of life scores were found to be higher in the group program. The concept of hope is considered an important psychological determinant of improvement, especially in chronic and recurrent conditions such as substance use disorder. Studies in adults with substance use disorder have shown that hope is strongly associated with life satisfaction, future orientation, and psychological resilience (39, 40). Similarly, it has been reported that increases in “state hope” and general well-being in clients during the treatment process rise regularly throughout the treatment and that this is an important part of the therapeutic process (41). In this context, our results suggest that group programs can not only reduce symptoms but also help individuals perceive life as more meaningful and valuable, while developing a more hopeful outlook on the future.

The higher cognitive flexibility scores found in the group program also point to the importance of cognitive processes in substance use disorders (42). Cognitive flexibility is associated with the capacity to develop alternative perspectives on challenging situations and to perceive the situation as more controllable. Individuals with high levels of cognitive flexibility have been shown to have lower levels of depression and anxiety, as well as more adaptable coping strategies (8; Fazeli et al.; 43). The opportunity for individuals to question their own thought patterns and develop alternative interpretations through case discussions, role-playing, and peer feedback in group interventions may have contributed to increased cognitive flexibility (44, 45).

On the other hand, this study did not observe a significant difference between individual and group programs in terms of addiction severity. There are several possible explanations for this. First, indicators based on usage patterns and diagnostic criteria, such as BAPI-Short, may reflect more stable and slowly changing characteristics over time; major differences in these indicators should not be expected in a short intervention period. Second, due to the cross-sectional design of the study, it is not known exactly at what baseline levels the participants arrived at which programs; that is, the groups may have been similar in terms of substance use severity even before the intervention. Meta-analyses examining the effects of group treatments on reducing substance use show that significant effects generally emerge with longer follow-up periods and repeated measurements (30). Therefore, future longitudinal designs are needed to evaluate the potential effects of the group program on substance use severity.

The significantly lower levels of depression and anxiety in the group program are consistent with results regarding the effects of group interventions on mood in substance use disorder treatment. Integrated group therapy studies have reported improvements in both substance use and mood symptoms (46, 47). One possible interpretation of these findings is that the group setting may be associated with therapeutic processes such as empathy, normalization, mutual sharing, and a sense of belonging. However, the present design does not allow us to determine whether these factors caused the observed between-group differences. In addition, studies conducted in the Turkish context have shown that increased treatment motivation and confidence-in-treatment are also among the important outcomes of group work (33).

From a clinical perspective, the findings suggest that group-based programs within probation settings are associated with more favorable psychosocial profiles in domains relevant to recovery, including hope, self-efficacy, social support, and cognitive flexibility. (48, 49). Numerous studies highlight that group programs can be more cost-effective than individual counseling, allow for the simultaneous service of more individuals, and improve both access to and continuation of treatment (30, 50, 51). Nevertheless, given the cross-sectional design, these associations should not be interpreted as evidence that group programs directly strengthen these resources or reduce long-term risks. Future longitudinal and randomized studies are needed before such conclusions can be made.

At the same time, this pattern should be interpreted cautiously considering possible bias. This comparison was based on naturally occurring probation groups rather than random assignments, and therefore, it is possible that participants in the group format were already more motivated, socially engaged, or psychologically resourced at baseline. Likewise, the exclusive use of self-report measures may have been particularly vulnerable to social desirability and impression-management tendencies in a probation context. These sources of bias may have contributed to the more favorable psychosocial profile observed in the group program.

An important practical implication of these findings concerns continuity of care after the probation period ends. These probation-based psychosocial programs are inherently time-limited, and therefore, gains observed in psychosocial domains such as hope, perceived social support, and self-efficacy may be difficult to sustain without ongoing recovery-oriented support. In this context, referral pathways from probation services to community-based self-help and mutual aid groups may represent an important continuation strategy. Mutual-help participation has been associated with stronger recovery support networks and improved longer-term recovery trajectories in the substance use literature. Recent evidence from Türkiye also suggests that self-help organizations may provide individuals with substance use disorders and their families with a meaningful recovery environment characterized by peer identification, information exchange, motivation for change, and sustained social connection. Therefore, establishing structured referral mechanisms from probation-based programs to community recovery resources may help bridge the gap between short-term institutional intervention and long-term recovery maintenance (37, 52, 53).

### 4.1 Limitations

This study has several limitations that should be considered when interpreting the findings. First, there are important methodological limitations. The study was cross-sectional, and participants’ psychosocial levels before entering the program were unknown. Therefore, the observed between-group differences may reflect pre-existing differences rather than the specific influence of program format. Given that the groups were naturally formed and participants were not randomly assigned, self-selection and administrative selection processes may have played a significant role. Participants referred to or retained in the group-based program may have differed from those in the individual format in terms of motivation for treatment, interpersonal comfort, communication skills, readiness for group participation, symptom burden, or overall psychosocial functioning.

Second, there are relevant measurement-related and analytical limitations. All outcome measures were based on self-report instruments, creating the possibility of social desirability, recall bias, response style effects, and shared method variance. These concerns may be particularly important in probation settings, where participants may perceive themselves as being evaluated within a legal-administrative context. In addition, although adjusted analyses were conducted, the observational and non-randomized nature of the comparison limits the extent to which statistical control can eliminate unmeasured confounding.

Third, there are important contextual limitations. Participants were recruited from a single probation office and the sample consisted predominantly of men; therefore, caution is needed when generalizing the findings to other probation regions, service structures, female individuals on probation, or broader treatment-seeking substance use disorder populations. Moreover, the psychosocial programs were evaluated as routine service formats rather than fully manualized research interventions, and some variation in implementation may have occurred across participants or sessions.

Fourth, the sample size was determined pragmatically based on the number of eligible individuals on probation available in the two program formats during the study period and who agreed to participate. Rather than being based on *a priori* random sampling from a wider probation population, the sample reflects feasible recruitment within a real-world probation setting. For this reason, the study should be considered exploratory and hypothesis-generating with respect to between-group psychosocial differences, and the findings should be interpreted with appropriate caution regarding generalizability.

Finally, the study did not evaluate longer-term outcomes such as relapse, recidivism, continued treatment engagement, or post-probation recovery status. Accordingly, the present findings should be interpreted as cross-sectional differences between two routine probation program formats rather than as evidence of differential intervention effects.

### 4.2 Future studies

Despite these limitations, the present study is one of the few studies comparing individual and group-based psychosocial programs among individuals on probation under Article 191 of the Turkish Penal Code across a broad range of psychosocial and mood-related variables. The finding that participants in the group program showed more favorable psychosocial profiles highlights the need for future studies using pre-post, longitudinal, and follow-up designs to determine whether these differences are sustained over time.

Future research should also incorporate baseline assessment before program entry, repeated outcome measurement, and longer-term follow-up indicators such as relapse, treatment continuation, recidivism, and post-probation psychosocial adjustment. Where feasible, randomized controlled designs or more rigorously matched quasi-experimental studies should be used to reduce selection bias and clarify whether observed differences are attributable to program format or to pre-existing participant characteristics. In addition, multi-center studies from different probation regions in Türkiye would improve external validity and help determine whether the present findings are specific to this institutional setting or generalizable across probation contexts. Finally, future studies should examine which specific components of group-based programs—such as peer interaction, emotional sharing, psychoeducation, or skills training—are most strongly associated with recovery-related psychosocial functioning.

## Data Availability

The raw data supporting the conclusions of this article will be made available by the authors, without undue reservation.
